# Editorial: Challenges in chronic thrombo-embolic pulmonary hypertension (CTEPH)

**DOI:** 10.3389/fmed.2023.1326429

**Published:** 2023-11-22

**Authors:** John David Aubert, Silvia Ulrich

**Affiliations:** ^1^Pulmonary Division, Lausanne University Hospital and University of Lausanne, Lausanne, Switzerland; ^2^Pulmonary Division, University Hospital Zurich, Zurich, Switzerland; ^3^University of Zurich, Zurich, Switzerland

**Keywords:** pulmonary hypertension, CTEPH, scintigraphy, CT scan, magnetic resonance, iodine mapping

Chronic thromboembolic pulmonary hypertension (CTEPH) is one of the five groups in the classification of pulmonary hypertension and is characterized by diagnostic imaging demonstrating perfusional defects of the pulmonary arterial circulation in addition to precapillary pulmonary hemodynamics. Until recently, the test of choice for making the diagnosis was the ventilation and perfusion lung scan (V/Q scan) with a gamma camera. A number of studies at the beginning of this century have established that V/Q scan was more sensitive and had a better negative predictive value than Computed Tomography Pulmonary Angiography (CTPA, Table 1) (1, 2).

**Table 1:** Imaging modalities for the diagnosis of CTEPH.

**Acronym**	**Procedure**	**Radiation exposure**	**Comments**
V/Q scan	Planar ventilation-perfusion scan with inhaled and perfused isotopes, detected through a gamma camera	+	Inhaled Xenon^133^ and Perfused Tc^99^ albumin macroaggregates
V/Q SPECT	Ventilation-perfusion scan coupled with single photon emission computed tomography	+	Allows visualization of the lung parenchyma
V/Q PET	Ventilation-perfusion recorded with positron emission tomography	+	Gallium^68^-labeled carbon particles and Ga^98^ albumin macroaggregates
CTPA	Computed tomography pulmonary angiography	++	Contraindicated with severe renal insufficiency.
DECT	Dual energy CT scan with iodine mapping	++	Contraindicated with severe renal insufficiency.
PREFUL-MRI	Phase-REsolved FUnctional Lung with magnetic resonance imaging	0	Cf. Duan et al.
DSPA	Digital subtraction pulmonary angiography	+++	Historical gold standard

Hence V/Q scan was positioned as a central screening tool in the diagnostic algorithm in guidelines and consensus statement for CTEPH (3–5).

However, several advances, both at the technical and at the image interpretation levels have challenged this view and an increasing number of PH centers rely nowadays on alternative imaging modalities for diagnosis. Among the many new imaging techniques for the pulmonary vasculature, single photon emission computed tomography (SPECT), has largely replaced conventional planar V/Q scan in clinical practice. The advantages are the simultaneous assessment of the lung parenchyma, together with an increased sensitivity and lower radiation exposure (6).

Another imaging technique that has emerged this last decade is Dual Energy CT Scan (DECT), which provides iodine maps as a surrogate marker of lung perfusion. DECT consists in performing a native CT scan followed by a conventional CTPA with contrast, then digitally subtracting the non-contrast CT from the CTPA to generate an iodine map. This technique allows the detection of perfusion defects even in the absence of visible morphological vascular alteration. Fawzy et al. are presenting such a case where subsegmental lung hypoperfusion was diagnosed on the subtracted reconstruction, while the vascular webs were barely visible.

In this topic specific series on CTEPH, Schüssler et al. have compared DECT with V/Q SPECT in 28 patients suspected of CTEPH. Invasive pulmonary angiography (PA) was used as the gold standard diagnostic procedure. The authors found that compared to PA, both accuracy and concordance for DECT were superior to V/Q SPECT in all patients as well as in the 18 patients with definitive CTEPH diagnosis. In addition, the mean radiation dose was significantly lower for DECT vs. V/Q SPECT. The limitation of this single center study is the low number of patients as, obviously, it would be difficult to consider performing all three imaging modalities for every patient in a large cohort. Another concern is the absence of patients with group 1 PAH, as a frequent differential diagnosis in the real life of a PH clinic is indeed to distinguish between idiopathic PAH and distal CTEPH. Finally, as for most imaging modalities, the radiologist's experience is key, and may not always reach the high level of expertise as obtained in specialists performing a dedicated study.

Magnetic Resonance Imaging (MRI) has also been used for CTEPH diagnosis and quantification. A comparable sensitivity to V/Q scan has been shown for dynamic contrast-enhanced MRI using Gadolinium (7, 8).

Recently, a new postprocessing technique known as phase-resolved functional lung (PREFUL) MRI has been developed, which improves the effective temporal resolution. Duan et al. have compared this technique with SPECT in 86 patients suspected of CTEPH. Compared to V/Q SPECT as the gold standard, the agreement of PREFUL-MRI was above 90% both at the patient and at the lobe level. This technique may be of interest for diagnosing CTEPH in patients with contrast agent allergies, patients with multiple short-term follow-up examinations, and patients who cannot hold their breath. It is often argued in review papers and medical societies guidelines that due to its high cost and low availability, MRI could not yet be recommended as a first line procedure. Although it is indeed a concern, it should be noted on the other hand that standard V/Q scan facility is no more available in many medium size hospitals, as it has been largely replaced by CTPA for the diagnosis of acute pulmonary embolism. Furthermore, several shortage episodes of radiolabelled gas (Xenon^133^) have occurred in the past years, jeopardizing the daily availability of this technique.

While the emergence of these new imaging facilities has the potential to improve the diagnosis of CTEPH, the non radiologist physician may be a bit confused by the technical aspects of the examination and the ever expanding number of acronyms. Furthermore, large prospective direct comparisons of these techniques remain scarce. It is therefore essential that each patient is discussed at a multidisciplinary meeting attended by the radiologist responsible for the examination, the lung specialist and the cardiologist, as well as the thoracic surgeon who will assess the operability of cases where CTEPH is confirmed (3).

## Author contributions

JA: Conceptualization, Writing—original draft. SU: Writing—review & editing.
